# Surgical audit of outcome of rhegmatogenous retinal detachment repair at Vitreoretinal unit JPMC in year 2014

**DOI:** 10.12669/pjms.321.9297

**Published:** 2016

**Authors:** Saifullah Tareen, Muhammad Ali Tahir, Alyscia Miriam Cheema

**Affiliations:** 1 Dr. Saifullah Tareen. FCPS, Department of Ophthalmology, Jinnah Post Graduate Medical Centre, Karachi, Pakistan; 2 Dr. Muhammad Ali Tahir. FCPS (Ophth), FCPS (Vitreoretina), Department of Ophthalmology, Jinnah Post Graduate Medical Centre, Karachi, Pakistan; 3 Dr. Alyscia Miriam Cheema. FCPS, FRCS, Department of Ophthalmology, Jinnah Post Graduate Medical Centre, Karachi, Pakistan

**Keywords:** Surgical audit, Rhegmatogenous retinal detachment, Removal of silicon oil

## Abstract

**Objective:**

To investigate the outcome of rhegmatogenous retinal detachment repair at Vitreoretinal unit of Jinnah Post Graduate Medical Centre Karachi in year 2014.

**Methods:**

One hundred and three eyes of one hundred and three patients, who underwent three ports parsplana vitrectomy + band + silicone oil, three ports pars plana vitrectomy + silicone oil, three ports pars plana vitrectomy + C3F8 for rhegmatogenous retinal detachment (RRD) repair, at Jinnah Post Graduate Medical Centre, were included in this observational prospective study. Parsplana vitrectomy was done using 23G vitrectomy system. Duration of study was one year. Removal of silicone oil (ROSO) was done on the basis of completely flat retina at least for eight weeks or because of complications due to silicone oil. Patients were followed up post operatively on day one and after one week and then at four weekly interval till the end of the study.

**Results:**

Anatomical success was achieved in 91 eyes (88.3%). However in 12 eyes (11.7%) retina redetached after removal of silicone oil. Functional success achievement of visual acuity of 3/60 or better was achieved in 85 (82.5%) of eyes post operatively after removal of silicone oil or absorption of gas C3F8 as the case may be.

**Conclusion:**

Re-detachment is common after removal of silicone oil and incidence of re-detachment is related to the degree of preoperative PVR and location of breaks. Re-detachment occurs more commonly if the breaks are inferiorly located as compared to the superior ones.

## INTRODUCTION

Retinal detachment is a major threat to sight in working age population. Retinal detachment (RD) is the separation of neurosensory retina (NSR) from the underlying retinal pigment epithelium (RPE).^1^ There are various types of retinal detachments amongst them the most commonly seen type is rhegmatogenous retinal detachment (RRD). It is characterized by the presence of a retinal break which is a defect involving whole thickness of neurosensory retina.^2^ Successful retinal detachment repair was initially described by Gonin in 1920 and advancements in technology have resulted in better results of surgery.^3^,^4^ This is a very debatable topic in retina community about which technique should be adopted to repair different types of retinal detachments. Understanding the basics and constraints of different treatment options is very important in improving visual results. Retinal attachment surgery can be successful only when there is no remaining traction between vitreous and retina and retinal breaks are properly sealed. Surgical methods commonly described to repair uncomplicated RRD are pneumatic retinopexy procedure, scleral buckling, and pars planavitrectomy. Scleral buckle alleviate vitreous and retina traction and causes the margins of retinal break to come close to underlying retinal pigment epithelium.^2^

Robert Machemer in 1970 modernized surgery with the launch of three ports pars planavitrectomy.^5^ Recent technological advancements in vitrectomy machines along with the use of silicone oil to treat complicated retinal detachments have resulted in better success level of retinal detachment surgery. The rationale of this study was to do surgical audit of postoperative success rate of RRD repair at department of Ophthalmology JPMC during year 2014.

## METHODS

One hundred and three eyes of one hundred and three patients, who underwent three ports parsplana vitrectomy + band + silicone oil, three ports pars planavitrectomy + silicone oil, three ports pars planavitrectomy + C3F8 for rhegmatogenous retinal detachment (RRD) repair, at Jinnah Post Graduate Medical Centre, were included in this observational prospective study. Twenty patients were lost to follow up and eight patients did not give consent so were not included in this study. Informed consent for the study and the procedures was taken from the patients who were enrolled for the study. The duration of study was one year from January 2014 to January 2015. Plan of removal of silicone oil (ROSO) was made on the basis of completely flat retina at least for eight weeks or because of complications due to silicone oil. Proformas containing detail of medical and ocular examinations were filled. All eyes underwent complete preoperative ocular examination and assessment of visual acuity. Preoperative data regarding age, sex, eye involved, details of first surgery i.e. parsplana vitrectomy using 23G vitrectomy system, encircling band, silicone oil injection (5000 CS) or use of C3F8 gas were recorded. Visual acuity, lens status was reviewed. One consultant performed all the surgeries. Oil was removed using passive method by oil-fluid exchange and later on fluid air exchange. While removal of silicon oil was done eye was assessed as to whether further laser or cryotherapy is required or not and if required was done peroperatively. After ROSO ports and conjunctiva were closed. Patients were followed up post operatively on day one and after one week and then at four weekly interval till the end of the study. At the end of the study the data was analyzed using SPSS version 16. Anatomical success was taken as flat retina till the last follow-up. Retinal redetachment with in twelve months of removal of silicone oil or absorption of C3F8 after PPV in eyes where C3F8 was used as endotemponade, was considered a failure. Functional success was taken as visual acuity of ≥ 3/60 at the last followup.

## RESULTS

One hundred and three eyes of one hundred and three patients attending the retina clinic at department of ophthalmology JPMC were included in this prospective study. Of these 62 were male, 41 female. The average age of patient was 42.92 years (range 17-75 years). Silicone oil was successfully removed from the eyes of 103 patients. The duration of silicone oil temponade ranged from two months to eighteen months however mean duration was six months. Mean post-operative IOP was 12.64mmHg and it ranged from 8mmHg to 30mmHg.

Out of 103 eyes operated for retinal detachment crystalline lens was present in 48 eyes, 45 eyes were pseudophakic and 10 eyes were aphakic. Forty seven eyes had breaks in inferior half of the retina, breaks were not found in 12 eyes preoperatively and rest of the eyes had breaks in superior half of the retina. Three basic techniques adopted for rhegmatogenous retinal detachment repair were PPV+ Scleral buckling + Silicon oil in 49 eyes, PPV + Silicon oil in 48 patients and PPV + Gas in 6 patients. The choice of surgical procedure was based on clinical evaluation and surgeon’s preference. In PPV + scleral buckling + silicone oil group out of 49 eyes after ROSO anatomical success was achieved in 44 eyes and in 5 eyes anatomical failure occurred. However in PPV + silicone oil group out of 48 eyes anatomical success was achieved in 41 eyes after ROSO and anatomical failure occurred in 7 eyes and in PPV + Gas group anatomical success was achieved in all the six eyes. Anatomical success was achieved in 91 eyes (88.3%) however in 12 eyes (11.7%) retina redetached after removal of silicon oil and were kept on list for redo VR surgery. Functional success was taken as achievement of visual acuity of 3/60 or better which was achieved in 85 (82.5%) of eyes post operatively after removal of silicon oil or absorption of C3F8 as the case may be.

**Table-I:** General Characteristics.

	Frequency	Percent
Total Eyes	103	100.0
Right	55	53.4
Left	48	46.6
*Gender*		
Male	62	60.2
Female	41	39.8
*Age in years*		
Mean ± S.D	42.9 ± 16.82	
Range	17 - 74	
*Pre-Operative Visual Acuity*		
Better (1/60 or 2/60)	17	16.5
Success (3/60 or 6/60)	11	10.7
CF	6	5.8
HM	66	64.1
PL+	3	2.9
*Method used for surgery*		
PPV + scleral buckling + silicon oil	49	47.6
PPV + silicon oil	48	46.6
PPV + Gas	6	5.8
*Intraocular pressure (mmHg) IOP*		
Mean ± S.D	12.6 ± 3.74	
Range	8 – 30	
*Status of the lens*		
Crystalline lens	48	46.6
Pseudophakic	45	43.7
Aphakic	10	9.7
*Location of break*		
Inferior	47	45.6
Superior	44	42.7
N/A	12	11.7
*PVR*		
PVR A present	7	6.8
PVR B present	53	51.5
PVR C present	43	41.7

**Fig.1:**
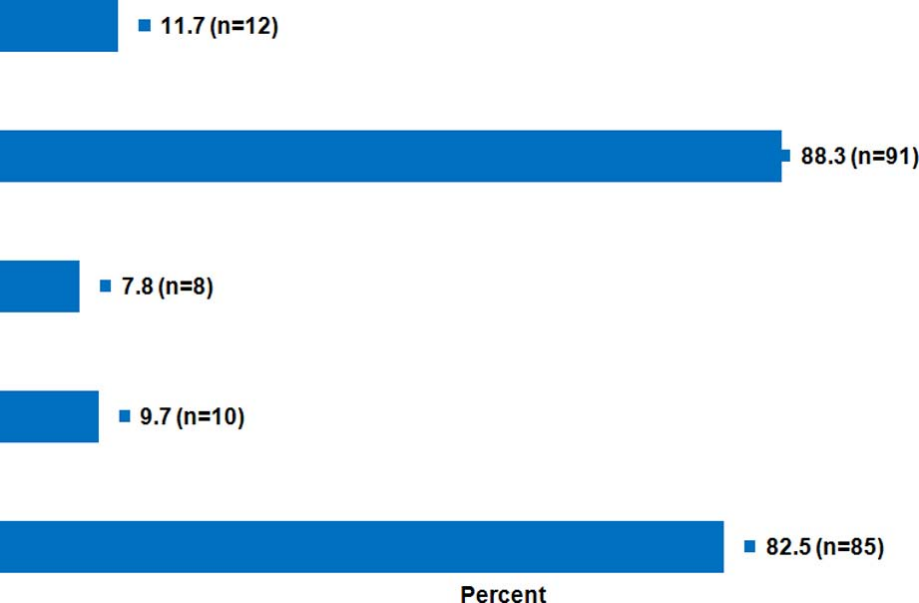
Postoperative outcome.

## DISSCUSSION

Complicated retinal detachments generally requires silicone for endotemponade. Silicone oil is non expansile liquid polymer. It is unmixable both in water and perfluorocarbon liquid and forms clear meniscus throughout intraoperative use. Viscosity of silicone oil is greater than gas however its surface tension and buoyancy are far less than that of gas.^6^ Silicone oil may have toxic effects however this issue is debatable.^7^ In the current study rate of retinal redetachment after ROSO or after absorption of gas varied according to the degree of PVR. In cases having preoperative PVR A all the seven cases did well and retina remained attached post operatively till last follow up. However out of 53 patients having preoperative retinal detachment with PVR B retina remained attached in 48(90.6%) of cases after (removal of silicone oil/ gas absorption which ever was used) and remained detached in 5 (9.4%) of cases. In a study conducted by Demir M et al. anatomical triumph was attained in 96% of cases^8^ with PVR B or less but their sample size was relatively smaller than in our study group this maybe one of the reason for statistical difference. We saw that success rate dropped in cases of PVR C and out of 43 cases 36(83.7%) of the patients had their retina finally successfully attached till last follow up and in remainder 7(16.3%) of cases retina remained detached inspite of Redo surgeries. In patients with PVR C along with inferior breaks preferred approach was PPV + scleral buckling + silicon oil however eyes in which PPV + silicon oil was done also did well and required more frequent retinotomies than the prior group.

According to some studies in cases of inferior retinal breaks scleral buckling in addition to vitrectomy supplement the less effective temponade at inferior retina.^9^-^12^ It does increase the operating time. However ideal buckling element in cases of complicated retinal detachment is shown to improve surgical results but some authors are of the opinion that by vitreous base shaving we can avoid use of buckling element.^13^-^16^ PVR was more commonly observed in cases with PPV + scleral buckling as compared to PPV alone in rhegmatogenous retinal detachments with inferior breaks according to some authors.^16^,^17^ Natarajan concluded that various authors are of the opinion that addition of buckling element increases the protection against re-detachment of retina after ROSO.^18^ Frequency of re-detachment after removal of silicon oil is reported to be between 0% and 32%.^19^,^20^ This variability may be due to difference in surgical technique, degree of preoperative PVR before surgery, sample size, duration of follow up after silicon oil removal and underlying disease processes. About 17.4% cases reported by Falkner^21^ re-detached after removal of silicon oil, which was very close to our study results of 16.3% of cases in PVR C subgroup. Scholda observed 20.5% cases of retinal detachment after silicon oil was removed.^22^ Pavlovic insisted that in case of completely attached retinas the risk of re-detachment is reduced after silicon oil removal.^23^

**Table-II:** Association of Variables with Anatomical success (Retina attached / detached).

Variables	No. of subject	Anatomical Success		P-value

		Retina attached	Retina detached	
Total Eyes	103	91 (88.3%)	12 (11.7%)	-
Right	55	55 (100.0%)	-	0.001
Left	48	36 (75.0%)	12 (25.0%)
*Gender*				
Male	62	53 (85.5%)	9 (14.5%)	0.265
Female	41	38 (92.7%)	3 (7.3%)
*Age in years*				
Up to 40 years	48	43 (89.6%)	5 (10.4%)	0.715
More than 40 years	55	48 (87.3%)	7 (12.7%)	
*Status of the lens*				
Crystalline lens	48	43 (89.6%)	5 (10.4%)	
Pseudophakic	45	38 (84.4%)	7 (15.6%)	0.358
Aphakic	10	10 (100.0%)	-	
*Method used for surgery*				
PPV + scleral buckling + silicon oil	49	44 (89.8%)	5 (10.2%)	
PPV + silicon oil	48	41 (85.4%)	7 (14.6%)	0.524
PPV + Gas	6	6 (100.0%)	-	
*Intraocular pressure (IOP) mmHg*				
< 12 mmHg	46	45 (97.8%)	1 (2.2%)	0.007
12 & above mmHg	57	46 (80.7%)	11 (19.3%)	
*PVR*				
PVR A	7	7 (100.0%)	-	0.355
PVR B	53	48 (90.6%)	5 (9.4%)	
PVR C	43	36 (83.7%)	7 (16.3%)	
*Location of break*				
Inferior	47	43 (91.5%)	4 (8.5%)	0.003
Superior	44	41 (93.2%)	3 (6.8%)	
N/A	12	7 (58.3%)	5 (41.7%)	

## CONCLUSION

Re-detachment is common after removal of silicon oil and incidence of re-detachment is related to the degree of preoperative PVR and location of breaks. Re-detachment occurs more commonly if the breaks are inferiorly located as compared to the superior ones.
